# Functional Polymeric Membranes with Antioxidant Properties for the Colorimetric Detection of Amines

**DOI:** 10.3390/s23229288

**Published:** 2023-11-20

**Authors:** Despoina Kossyvaki, Matteo Bustreo, Marco Contardi, Athanassia Athanassiou, Despina Fragouli

**Affiliations:** 1 Smart Materials, Istituto Italiano di Tecnologia, Via Morego 30, 16163 Genova, Italy; despoina.kossyvaki@iit.it (D.K.); marco.contardi@iit.it (M.C.); athanassia.athanassiou@iit.it (A.A.); 2 Dipartimento di Informatica Bioingegneria, Robotica e Ingegneria dei Sistemi (DIBRIS), Università degli Studi di Genova, Via Opera Pia 13, 16145 Genova, Italy; 3 Pattern Analysis and Computer Vision, Istituto Italiano di Tecnologia, Via Enrico Melen 83, 16152 Genova, Italy

**Keywords:** porous electrospun fibers, curcumin, gas sensing, pH indicators, antioxidant, trimethylamine, cadaverine, putrescine, spermidine, histamine

## Abstract

Herein, the ability of highly porous colorimetric indicators to sense volatile and biogenic amine vapors in real time is presented. Curcumin-loaded polycaprolactone porous fiber mats are exposed to various concentrations of off-flavor compounds such as the volatile amine trimethylamine, and the biogenic amines cadaverine, putrescine, spermidine, and histamine, in order to investigate their colorimetric response. CIELAB color space analysis demonstrates that the porous fiber mats can detect the amine vapors, showing a distinct color change in the presence of down to 2.1 ppm of trimethylamine and ca. 11.0 ppm of biogenic amines, surpassing the limit of visual perception in just a few seconds. Moreover, the color changes are reversible either spontaneously, in the case of the volatile amines, or in an assisted way, through interactions with an acidic environment, in the case of the biogenic amines, enabling the use of the same indicator several times. Finally, yet importantly, the strong antioxidant activity of the curcumin-loaded fibers is successfully demonstrated through DPPH^●^ and ABTS^●^ radical scavenging assays. Through such a detailed study, we prove that the developed porous mats can be successfully established as a reusable smart system in applications where the rapid detection of alkaline vapors and/or the antioxidant activity are essential, such as food packaging, biomedicine, and environmental protection.

## 1. Introduction

Amines are nitrogenous, organic compounds that are formed with the decarboxylation of amino acids, or amination and transamination of aldehydes or ketones by microorganisms [1]. These low molecular weight bases can be found in a wide variety of environments. For instance, they are normally present in small concentrations in the tissues of bodies with biological activity [2] and, consequently, in protein-based food, with their concentration increasing due to tissue decay or food deterioration [3]. Moreover, they are used in drugs [4], for gas treatment [5], and in epoxy resins as curing agents [6]. Regardless of their origin, in large concentrations, amines can cause skin irritations [7] and severe symptoms upon ingestion, ranging from nausea and headaches to heart problems, and even death [8,9,10,11]. For these reasons, effective methods to trace their presence are needed. 

Amines can increase the pH of the environment in which they are found due to their alkaline nature, therefore, their tracing can be succeeded through pH monitoring using indicators [12]. The most popular pH indicators are the colorimetric ones, which provide qualitative or semi-quantitative information about the studied environment through visual colorimetric changes or through comparison with standard references [13]. To do so, molecules able to present a different hue depending on the pH of the environment have been utilized as the principal components of these indicators, with natural molecules being the most prevalent preference, mainly due to their biocompatible and sustainable nature [14,15,16,17,18,19,20,21].

A popular natural pH-sensitive molecule is curcumin, or (1,7-bis(4-hydroxy-3-methoxyphenyl)-1,6-heptadiene-3,5-dione). It is a linear polyphenolic compound and natural food pigment with a characteristic yellow-orange color, extracted from the rhizome of turmeric (*Curcuma longa* and *Curcuma domestica*) [22]. As presented in Figure S1 of the Supporting Information, curcumin consists of two o-methoxy phenolic groups connected by a seven carbon linker, an α,β-unsaturated β-diketo moiety [23]. The diketo group exhibits keto–enol tautomerism and can exist in different types of conformers depending on the nature of the environment with which it interacts. In acidic and neutral environments (pH ≤ 8), curcumin has a yellow color, while at pH > 8 it becomes reddish-brown. These changes can be attributed to the reversible structural transformation of the molecule. In particular, the keto form of curcumin dominates in acidic and neutral media, whereas the enol form predominates at pH values above 8 [24,25]. In the transition of curcumin from acidic to basic medium, the phenolic hydroxyl group deprotonates to form the phenoxide anion causing the color change from orange to reddish-brown [23,26]. Curcumin is also widely known for its pharmacological activity and therapeutic properties, especially for its antioxidant properties [27]. Indeed, there are plenty of studies dedicated to its ability to scavenge the free radicals in various systems [22,28,29,30], a very important property for many applications such as food packaging and wound healing, due to the harmful role of the free radicals in foods and biological systems, respectively [31,32,33].

Due to its aforementioned properties, curcumin has been widely used to fabricate materials for various scopes, including antioxidant systems and pH-indicating platforms [22,34,35,36]. Specifically for the latter, recent research is oriented towards the introduction of curcumin to porous materials with high available surface area such as foams or fibrous mats [14,37,38,39,40,41,42,43], and, most recently, porous fibers [13]. The latter structure is more likely to guarantee an efficient interaction between the pH-sensitive compound and the surrounding environment of the material, presenting faster responses and higher sensitivity with respect to films of the same composition [44]. However, most of the already existing studies just present either an evaluation of the pH-induced color changes of the indicators upon their immersion into buffer solutions [43,45,46], or generic tests with vapors of fixed concentrations. Further investigation of the material’s interaction with specific microbial metabolites [47], the detection limit and/or the response time [39,48,49,50,51] or color reversibility studies are rarely presented [48,52,53]. In addition, the investigation of the interaction with the polymeric indicators should include studies with microbial metabolites, i.e., various types of amines, in order to understand their sensitivity, their potential selective responsiveness, and their capability to recover their color after the exposure [54].

Hence, in this work, the responsive capability of polycaprolactone-curcumin porous fibers, fabricated through non-solvent induced phase separation during electrospinning [13,55,56], to volatile and biogenic amine vapors, in terms of responsivity, sensitivity and reusability, is investigated. In particular, the ability of the curcumin-based indicators to detect the presence of the amine vapors, along with their reversibility and reusability are thoroughly investigated through detailed vapor exposure tests. Moreover, the radical scavenging activity of the curcumin-loaded material is studied, verifying that the curcumin’s antioxidant activity is maintained also after its embedment to the polymeric material. The capability of the porous material to present rapid and visually perceivable color changes upon exposure to small concentrations of the studied amines, and its antioxidant activity make it an ideal candidate for various applications, in smart packaging, biomedicine, and other fields, either for the immediate acquisition of information regarding the environmental quality, or as an antioxidant system. It is of note that the fast and easy one-step fabrication process of the mats with the direct incorporation of curcumin, the availability of the latter to interact with both the amine vapors and the free radicals, along with the lack of elevated temperatures during their production, contribute to overcoming the obstacles for the scale-up of such materials [22], bringing them one step closer to the market.

## 2. Materials and Methods

### 2.1. Materials

Curcumin from *C. longa* (turmeric) powder, polycaprolactone (PCL) (M_n_ ≈ 80,000 Da), chloroform (≥99.8%), acetone (≥99.5%), ethanol (96%), dimethyl sulfoxide (DMSO) (≥99.5%), 2,2′-azino-bis(3-ethylbenzo-thiazoline-6-sulfonic acid) diammonium salt (ABTS), 2,2-diphenyl-1-picrylhydrazyl (DPPH), cadaverine (CAD), (95%), histamine (HIS), (≥97.0%), trimethylamine (TMA), (~43–49 wt% in H_2_O (T)), spermidine (SPE), (≤100%), putrescine (PUT) (analytical standard), and hydrochloric acid (HCl) (30%) were purchased by Sigma Aldrich and used without further purification. Deionized water was obtained from a Milli-Q Advantage A10 ultrapure water purification system.

### 2.2. Fabrication of the Fibers

The fabrication of the porous fibers was performed by means of vertical electrospinning (Figure S2a of Supporting Information) through the non-solvent induced phase separation. This method is based on the great difference in the boiling points of the solvents involved, and on the fact that the solvent with the high boiling point is a non-solvent for the polymer [56]. During the electrospinning, the good polymer solvent(s) will be the first one(s) to evaporate, and as a result, the non-solvent with the high boiling point will remain on and in the electrospun fibers, with its subsequent slower evaporation creating holes on and in them, the so-called pores (Figure S2b of Supporting Information). Hence, according to our previous work [13], electrospinning solutions were prepared by combining PCL (15.0% *w*/*v* with respect to its solvents) with curcumin (0.5 and 1.0 wt% with respect to the polymer), in a mixture of chloroform (boiling point ≈ 61.2 °C), acetone (boiling point ≈ 56.0 °C) (7:3 chloroform:acetone, volume ratio)—acting as the PCL’s solvents—and the PCL non-solvent DMSO (boiling point ≈ 189.0 °C, 9.0% *v*/*v* with respect to the final solvent mixture). Such solutions were stirred at room temperature (RT) until the complete dissolution of PCL and curcumin, and were subsequently electrospun with a vertical electrospinning system using the following optimized parameters: flow rate: 3 mL/h; applied voltage: 17 kV; distance of the aluminum collector from the spinneret tip: 31 cm at RT (ca. 21–23 °C) and relative humidity (RH): 53–57%. Following this process, mats of pure PCL, and PCL-curcumin 0.5 wt% and 1.0 wt% were formed and named as PCL, PCLCU05 and PCLCU1, respectively (Figure S3 of Supporting Information). The complete characterization of the morphology, porosity and surface area, together with their chemical, thermal, wetting, and mechanical properties can be found in our previously published work [13].

### 2.3. Antioxidant Activity: DPPH^●^ and ABTS^●^ Free Radical Scavenging Assays

The antioxidant activity of the fibers was investigated by the standard DPPH^●^ free radical scavenging assay and by the ABTS^●+^ radical cation scavenging assay. For the DPPH^●^ assay, pieces of PCLCU1 mats were placed in polystyrene (PS) cuvettes in a concentration 20% *w*/*v* with respect to 0.2 mM solution of DPPH^●^ radical in ethanol under dark, at RT for 24 h. The decrease in the radical signal absorption over time was monitored at 528 nm using a Cary 300 Scan UV-Visible spectrophotometer. A sample with only DPPH^●^ solution was used as control, to ensure the reagent stability. The radical scavenging activity (RSA) was expressed as the inhibition percentage of the free radical of the samples and was calculated using Equation (1):

(1) $$RSA\left(\%\right)=\left[\frac{{\left(A\right)}_{control}-{\left(A\right)}_{sample}}{{\left(A\right)}_{control}}\right]\times 100$$

where (A)_control_ is the absorption of the control sample and (A)_sample_ is the absorption of the sample at 528 nm at different time points [57,58,59].

The ABTS radical cation (ABTS^●+^) was generated by the reaction between 7 mM ABTS aqueous solution and 2.45 mM potassium persulfate aqueous solution. The reaction took place in the dark at RT for 12–16 h. ABTS and potassium persulfate react stoichiometrically at a ratio of 1:0.5, resulting in the incomplete oxidation of the ABTS. The oxidation of the ABTS starts immediately, but the absorbance does not become maximal and stable until some hours are elapsed [60]. The ABTS^●+^ solution was then diluted with water to obtain a starting absorbance of 1.2 arbitrary units at 734 nm. Right after the dilution, pieces of PCLCU1 mats were placed in PS cuvettes in a 20% *w*/*v* concentration with respect to the diluted ABTS^●+^ solution, and left under dark at RT for 24 h. The evolution of the decrease in the absorbance was measured at 734 nm by using a Cary 300 Scan UV-Visible spectrophotometer. A sample with only ABTS^●+^ solution was used as control, to ensure the stability of the solution. The RSA of the samples was expressed as the inhibition percentage of the free radical of the samples and was calculated by using the Equation (1).

It is of note that the two antioxidant tests were also made for samples loaded with less curcumin (PCLCU05), following the same protocols. All measurements were performed in triplicates.

### 2.4. Interaction of the Fibrous Mats with Amines

*CIELAB color space analysis.* For studying the colorimetric variation of the fibers, we used the CIELAB color space, a three-dimensional space designed for producing a color representation that mimics the human color perception. It consists of three components related to the lightness of the color (L), the hue position between red and green (a) and the hue position between yellow and blue (b). Given two CIELAB sample coordinates ($L_{1},a_{1},b_{1})$ and ($L_{2},a_{2},b_{2})$ describing the average color of the indicators extracted from different frames of videos or photos, their Euclidian distance (dE) provides an approximated quantitative measure of the human perceived color difference [61], which can therefore be calculated using the following Equation (2):

(2) $$dE=\sqrt{\left[{\left(L_{1}-L_{2}\right)}^{2}+{\left(a_{1}-a_{2}\right)}^{2}+{\left(b_{1}-b_{2}\right)}^{2}\right]}$$

When dE ≥ 5, the color difference is complete and clearly perceivable, while when it is dE ≥ 3.5, the color difference can be noticed by inexperienced observers [62]. 

The images of the mats have been acquired in RGB color coordinates using a reflex camera (Canon EOS 5D Mark II) and converted to CIELAB color coordinates in order to exploit the described space properties. During color conversion, the CIE standard illuminant D65 was used as a reference white point, theoretically corresponding to a color temperature of 6504 K.

*Response kinetics.* The evaporation of TMA is rather fast due to its high volatility, whereas biogenic amines (BAs), which have much higher boiling points than the ones of volatile amines (VAs) (Table S1 of Supporting Information) present a much slower evaporation at RT. For this reason, experiments were conducted in RT in the case of TMA, and on a hot plate set at 30 °C in the case of BAs. Unless differently stated, all experiments were performed in a sealed homemade cell of volume ≈ 11 cm^3^ equipped with two glass columns in the case of TMA and one glass column in the case of BAs, where PCLCU1 mats (thickness, 0.020 ± 0.002 mm; dimensions, 1.0 cm × 0.5 cm; and weight, 0.58 ± 0.09 mg) were fixed (Figure S4 of Supporting Information). Subsequently, droplets of 7 μL of TMA, CAD, SPE, PUT, or HIS aqueous solutions of various concentrations were separately placed in the cell through a tube that was sealed immediately after the droplet deposition, and left until their complete evaporation. 

In order to follow the color evolution of the mats, two different strategies were followed for the two categories of amines. In the case of TMA, 8-minute-long videos of 25 frames/s were recorded; in the first 4 min, the transition from yellow to red was followed, whereas after the 4 min, the cell was opened and the return from red to yellow was observed. In the case of BAs, videos of 25 frames/s were captured, except for the case of HIS, for which 1 frame was captured every 30 s for ca. 16 h due to its much slower evaporation. Since the color changes induced by the BAs are not spontaneously reversible (Figure S5 of Supporting Information), only the transition from yellow to red was followed. All experiments were conducted in triplicates. Videos and images were analyzed converting the relevant portion of the acquired images to CIELAB color coordinates. Subsequently, the dE with respect to the color coordinate at time 0 s has been plotted to graphs, showing the time evolution of the mean value of dE in each case. 

For the analysis of the dynamic response behavior of the porous indicators in the presence of the TMA and the BAs, the sigmoid mathematical model Boltzmann [63] (Equation (3)), was selected as the optimal fitting model:

(3) $$dE={dE}_{max}+\frac{{dE}_{0}-{dE}_{max}}{1+e^{\frac{t-t_{0}}{d_{t}}}}$$

where dE_0_ and dE_max_ are the minimum and maximum dE values, t_0_ is the inflection point (i.e., the time at which the dE reaches the 50% of the maximum value), and d_t_ is a time constant. The slope of the apparent growth of the dE that indicates the rate of the color change was calculated through the fitting parameters using Equation (4):

(4) $$slope=\frac{{dE}_{max}-{dE}_{0}}{4\times d_{t}}$$

It should be mentioned that the obtained data were also fitted using the pseudo-first order kinetics equation ($dE={dE}_{max}\times (1-e^{\left(-k\times t\right)})$) where k is the reaction rate constant (1/s), and t is in s [64]. The adjusted R-square indicated the suitability of the fitting only for the VAs (TMA, Figure S6 of Supporting Information). Therefore, for comparison reasons, we decided to explore the kinetics behavior and compare the data, using the same fitting model (Boltzmann) for all cases. 

*Detection limit.* For the investigation of the color change behavior of the mats towards different concentrations of VAs, the samples were exposed to 244.8 ppm, 122.4 ppm, 81.6 ppm, 48.9 ppm, 22.3 ppm, and 11.7 ppm of TMA vapors. In the case of BAs, the samples were exposed to BA vapors with concentrations ranging from 527.8 ppm to 11.5 ppm (Table S2 of Supporting Information). In all cases, the vapors were generated upon the evaporation of 7 μL droplets of aqueous solutions containing the amines in various concentrations—adjusted in order to result to the abovementioned vapor concentrations—inserted in the sealed homemade cell containing the sample. Subsequently, images were captured every 30 s for 2 min or for ca. 16 h for TMA and BAs, respectively, monitoring the color change in the sample due to the formation of the amine vapors in the cell. The experiments were conducted in triplicates in the case of TMA and in duplicates for the BAs, and analyzed by converting the acquired images to CIELAB color coordinates.

*Color reversibility.* Although in the case of the VAs the color of the porous indicator recovers spontaneously after the opening of the cell [13], in the case of BAs the color reversibility does not occur spontaneously (Figure S5 of Supporting Information), but in an assisted way. Specifically, using CAD as a representative BA, and after the exposure of the PCLCU1 mat to 527.8 ppm of CAD vapors, three *yellow-red-yellow* cycles were performed. Right after their exposure to CAD, the mats were left in atmospheric conditions at RT for 24 h in the dark, in order to reassure that they did not return to their initial color spontaneously. Subsequently, they were dipped into an aqueous solution of HCl (1 M) and their color modification was monitored. As a control experiment, the color evolution of another CAD-exposed-mat subjected to ambient conditions and without any exposure to HCl was also studied. The chemical composition and the possible chemical interactions of the CAD-exposed samples, with, and without acid bath were studied with a Fourier Transform Infrared (FTIR) spectrometer (Vertex 70v, Bruker (Billerica, MA, USA)), equipped with an attenuated total reflectance (ATR) accessory (MIRacle ATR, PIKE Technologies, Fitchburg, WI, USA) using a diamond crystal. All spectra were recorded in the range of 4000−600 cm^−1^, with a resolution of 4 cm^−1^, accumulating 64 scans.

After the verification of the reversibility with the assistance of HCl in the liquid phase, the color recovery was also evaluated in the presence of HCl vapors, generated by a drop of 55 μL of 1 M HCl aqueous solution, performing three *yellow-red-yellow* cycles. For this experiment, two identical homemade cells were used for the basic and acid environment: after the exposure to CAD, the fixed mat was transferred to the second cell for the HCl exposure. Subsequently, after the color recovery, the mat was transferred back to the first cell for the re-exposure to CAD, and so on. To reassure that the color of the sample is not influenced by possible base and acid remnants, the cells after the exposure were cleaned with water and glassware soap. The color difference between the original CIELAB coordinates of the mat and the CIELAB sample coordinates after the amine and acid exposure of each cycle was analyzed.

## 3. Results and Discussion

### 3.1. Antioxidant Activity

Curcumin is an extremely potent lipid soluble antioxidant, and, as a phenolic compound, it presents two different mechanisms of antioxidant activity. The first mechanism includes the neutralization of the free radical by hydrogen atom transfer from the antioxidant, while in the second one, the free radical receives an electron from the antioxidant and becomes a radical cation, which could react with another antioxidant molecule [65,66]. 

Curcumin’s radical scavenging activity has been previously studied using the DPPH^●^ assay, usually used for hydrophobic compounds, and ABTS^●^ which has been used for both hydrophilic and lipophilic systems [27,31,67]. However, a recent study revealed that the ABTS^●^ assay is more appropriate than the DPPH^●^ when applied to a variety of plant foods containing, among others, lipophilic and high-pigmented antioxidant compounds [68]. In order to verify these findings, we decided to investigate the radical scavenging activity of the fiber mats with both DPPH^●^ and ABTS^●^ assays.

**Figure 1:**
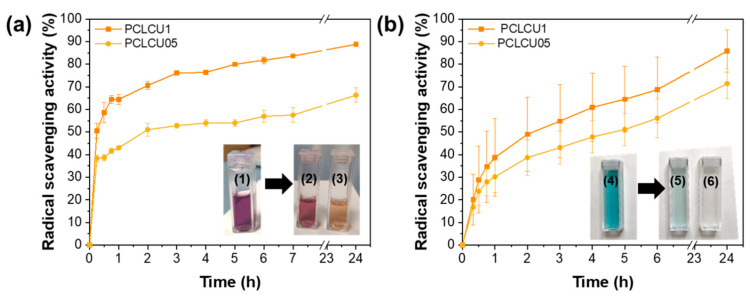
The radical scavenging activity of the PCLCU05 and PCLCU1 fiber mats with (**a**) DPPH^●^ and (**b**) ABTS^●^ radical scavenging assays. In the inset of (**a**) the DPPH^●^ solution (1) before and after the 24 h experiment with (2) PCLCU05 and (3) PCLCU1. In the inset of (**b**) the ABTS^●^ solution (4) before, and after the 24 h experiment with (5) PCLCU05 and (6) PCLCU1.

As shown in Figure 1, in both cases the developed mats demonstrate a strong antioxidant activity, depended on the concentration of curcumin in the composite and on the interaction time. This is verified also from the change in the color of the DPPH^●^ reagent solution from purple to pinkish in the case of PCLCU05 and to yellow in the case of PCLCU1, and from the discoloration of the ABTS^●^ reagent, that becomes more evident in the case of PCLCU1, verifying that the antioxidant activity becomes stronger as the concentration of curcumin increases. 

Using the DPPH^●^ assay, an RSA of 66.3 ± 3.2% for the PCLCU05 and 88.8 ± 0.7% for the PCLCU1 was reached, after 24 h of interaction, with values reaching almost 40.0% in the first hour of exposure of the fiber mats to the reagent. Similar results were obtained also with the ABTS^●^ assay, with final RSA values of 71.4 ± 6.6% and 85.8 ± 9.4% for the PCLCU05 and PCLCU1, respectively (Figure 1b). In contrary to other studies where either the antioxidant activity is tested exclusively using the extract of the solid antioxidant composite [58,69,70], or the tested materials presented different radical scavenging activities when tested with different assays [71,72], in our case, we used directly the solid material (PCLCU05 and PCLCU1) in both assays, obtaining similar RSA final values. However, in the case of ABTS^●^, the process is slightly slower, most likely because curcumin is a lipophilic molecule. Therefore, the curcumin does not get released into the aqueous medium due to its hydrophobicity, but the antioxidant activity happens due to the contact of the whole mat with the ABTS^●^ solution through the electron donation to the free radical [2]. In fact, the higher standard deviations of the RSA mean values of Figure 1b, indicate the expected uncertainty of the number of the exposed curcumin molecules of the nanocomposite to the free radicals. On the other hand, since the ethanoic environment of DPPH^●^ is favorable for the release of curcumin, more curcumin is available in the reagent environment inducing an effective scavenging activity by hydrogen donation, mainly from the central active CH_2_ group of the keto form of curcumin and hydrogen abstraction from the phenolic group, at a shorter period of time, and the system reaches an RSA plateau value faster (Figure 1). 

Nonetheless, as already mentioned, both systems present similar final RSA values, something that can be attributed to the material’s structure and high porosity [13], a characteristic that makes possible the more direct interaction of both DPPH^●^ and ABTS^●^ reagents with the curcumin entrapped it the polymer matrix. Therefore, it is proved that the antioxidant activity of the herein presented curcumin composite is effective in both aqueous and ethanoic environments. It is of note that the PCL polymer does not have any antioxidant activity [73].

### 3.2. Interactions with the Amine Vapors

In our previous work [13] we demonstrated the importance of the high porosity on the responsiveness and sensitivity of the curcumin-based colorimetric indicators through the comparative study with non-porous equivalent fiber mats, using dimethylamine (DMA) vapors as a VA model system. Since DMA is a base, it is a better proton acceptor than water, and once they are mixed together DMA will abstract a proton from water and will form the conjugate acid dimethylammonium ion, and the conjugate base hydroxide anion [74] (5):

(5) $${\left({CH}_{3}\right)}_{2}NH+H_{2}O⇌{\left({CH}_{3}\right)}_{2}{{NH}_{2}}^{+}+{OH}^{-}$$

Once the DMA vapors make contact with the PCLCU1, the presence of OH^−^ causes the deprotonation of curcumin’s phenolic hydroxyl group and the formation of the phenoxide anion, and this optically results in the transformation of the material’s color from yellow to reddish/brown [23,74]. The formation of the enolate double bond extends the conjugation system of curcumin’s structure, resulting in a substantial bathochromic shift in the visible spectrum [75], and, therefore, in the color change in the PCLCU1 composite. Likewise, the reaction of curcumin with other amines is expected to follow a similar mechanism. 

**Figure 2:**
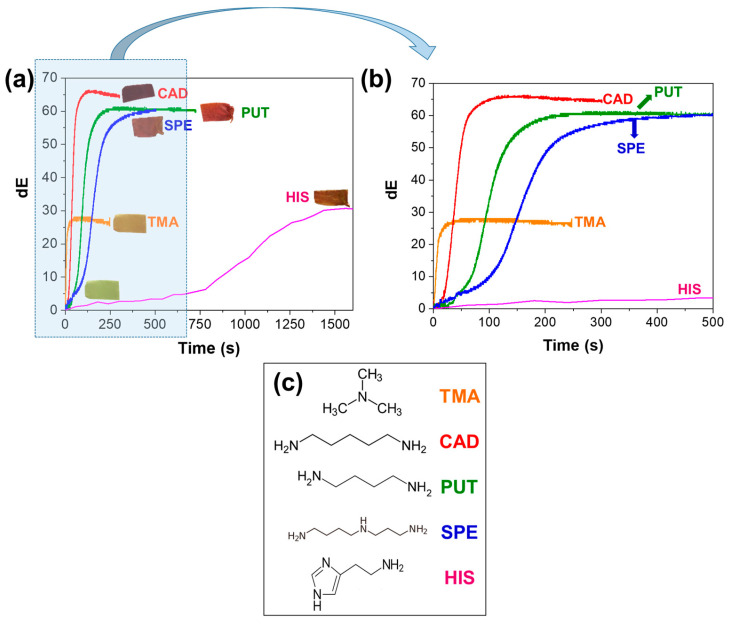
(**a**) The CIELAB color space analysis kinetics curves of the color evolution of the PCLCU1 mat during the exposure to the amines, and (**b**) the kinetics in the first 500 s in more detail. (**c**) The structures of the amines along with their names. The color of the amine names corresponds to the color of the curves.

To prove this, we investigate the responsiveness of the porous mats toward another VA, i.e., the TMA vapors formed by the evaporation of a TMA aqueous droplet (244.8 ppm) at RT in a closed system. As shown in Figure 2a, the CIELAB color space analysis shows that the exposure of the PCLCU1 to the vapors results to its rapid color change, from yellow to orange. More specifically, the dE of the TMA-exposed sample was able to reach a maximum dE value of ca. 29 in few tens of seconds. Additionally, the material reached the dE = 5, value that indicates that the color change can be perceivable by the users, in just 2.8 ± 0.5 s (Table S2 of Supporting Information).

When the porous material was exposed to CAD, PUT, SPE, and HIS vapors generated by the disposed droplets (527.8 ppm, 504.0 ppm, 588.6 ppm, and 689.2 ppm, respectively) the color change behavior varied depending on the type of amines studied. As presented in Figure 2a,b, and in Table S2 of Supporting Information, the time needed to reach a color change that can be perceivable by the users (dE = 5) is 2.5 ± 2.0 s for CAD, 22.0 ± 7.9 s for PUT, 24.9 ± 6.8 s for SPE, and 1080.0 ± 360.0 s for HIS. 

In most of the cases studied, the color response-time curve of the indicators shows a typical time-dependent sigmoid trend (Figure 2b). The sigmoid curve displays a shape, which can be divided into three distinct periods, the induction period (I), growth period (II), and stable period (III) (Supporting Information Figure S7). This behavior can be well-described by the Boltzmann equation (Equation (3)) with the fitting curve not always presenting a standard symmetrical shape, but also a right-skewed or a left-skewed type (Figure S6b–e, according to Figure S7 of Supporting Information) [76]. As shown in Figure 2b and in Figure S8 of Supporting Information, the induction period is apparently very short when the sample is exposed to TMA or CAD vapors, while it becomes greater for the other types of BAs, with the highest duration observed in the presence of HIS. This clearly indicates that the interaction of the material with the amine vapors depends on the type of the amines, and will be discussed in detail below. 

Through the kinetics analysis using Equation (3), and as can be shown in Table S2 and Figure 2, the inflection point t_0_, i.e., the time needed to reach 50% of the dE_max_, differs significantly depended on the type of amines. In the case of TMA, t_0_ is the smaller one indicating the most rapid response, while the slowest one is for HIS (t_0_: 13.2 ± 5.7 s for TMA vs. 44.1 ± 9.2 s for CAD, 84.9 ± 8.8 s for PUT, 117.0 ± 26.0 s for SPE, and 8307.6 ± 6541.4 s for HIS). Moreover, the color change rate was calculated to be 2.18 s^−1^ for the exposure to TMA, 1.75 s^−1^ for CAD, 0.66 s^−1^ for PUT, 0.42 s^−1^ for SPE, and 0.01 s^−1^ for HIS. It should be underlined that the experiment of TMA was conducted at RT and the TMA concentration in the deposited droplet was much lower compared to the BAs. In the case of evaporation temperature of 30 °C of a TMA droplet with a higher concentration (here we used 244.8 ppm of TMA vs. 527.8 ppm of CAD), the color change rate is expected to be even faster.

**Figure 3:**
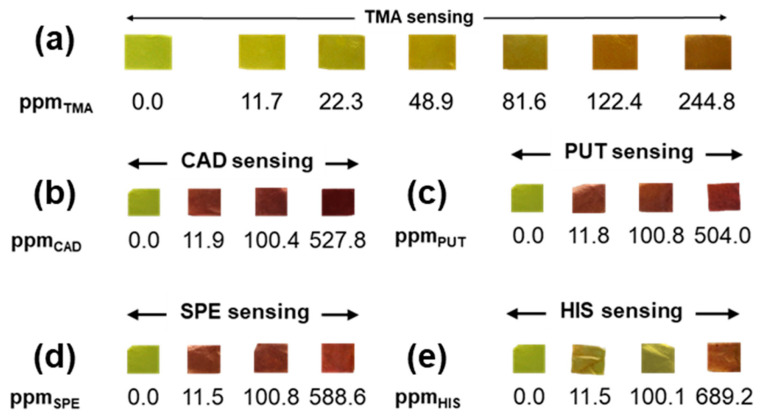
The color of the PCLCU1 upon exposure to each concentration of (**a**) TMA, (**b**) CAD, (**c**) PUT, (**d**) SPE, and (**e**) HIS.

For all of the TMA concentrations studied, ranging from 11.7 to 244.8 ppm in the liquid droplet (Figure 3a, Figure S9 of the Supporting Information), the PCLCU1 effectively changes color, reaching the maximum value in ca. 1 min for all cases. As shown in Figure S9 of the Supporting Information, a linear dependence of the dE_max_ to the TMA concentrations is observed, indicating a direct interaction of the curcumin molecules englobed in the mat, with the TMA vapors. Thus, from the linear fitting analysis of these data, the precise value of the minimum TMA concentration that can modify in a sufficient way the color of the indicator so as to be visually perceivable (dE = 5) is calculated to be 0.9 ppm (Note S1).

As for lower concentrations of the BAs, from Figure 3b–e and Table S2 of the Supporting Information, it can be noticed that PCLCU1 presents a dE_max_ well above 5 for all amine concentrations studied, down to 11.0 ppm. This demonstrates that the developed material can successfully trace all BAs, even of low concentrations. It can be noticed, though, a change in the time that the mats need to reach their maximum color difference, and to obtain a dE ≥ 5. As a general pattern, it seems that the lower the concentration of the amine, the lower the final dE_max_ that the material can reach and the more the time needed to reach it. 

More specifically, as shown in Figure S10 Supporting Information, depending on the concentration of amines, the induction period increases as we move toward lower BAs concentrations. For instance, in the case of CAD, an almost negligible induction period is noted when the composite mats are exposed to 527.8 ppm (Figure 2b and Figure S10a of Supporting Information), while the induction period becomes significantly longer in the case of the minimum concentration. This is attributed to the fact that the selected conditions reflect different relative ratios of curcumin and exposed amine vapors in the three different experimental conditions. Decreasing the concentration of the selected amine solution while the curcumin amount in the colorimetric indicator remains constant slows down the response rate and the dE_max_. A lower amount of amines in the droplet produces lower amounts of OH^−^ necessary for the curcumin’s color transformation, while the original color of the curcumin would disturb the appearance of the color change leading to the hysteresis phenomenon [76].

The difference in the reaction times of the material for the VAs and BAs vapors is strongly related to the type, size, structure (Figure 2c) and consequently to the chemical properties of the amine molecules (Table S1 of Supporting Information). In particular, the reactivity toward vapor-phase BAs is related to the vapor pressure (volatility), with PUT and CAD to have vapor pressures of 0.35 kPa and 0.13 kPa at 25 °C, respectively, which are significantly lower than the one of TMA (228.85 kPa) and therefore they appear to be less reactive at RT, justifying our choice to work at higher temperatures in the case of BAs [77]. On the contrary, TMA has the highest vapor pressure, and therefore it produces the highest concentration of vapors in the same space over the same time compared to the other amines presented herein, thus provoking the fastest colorimetric response. After TMA, CAD vapors react faster with the PCLCU1 with respect to PUT. According to the Table S1 of Supporting Information, we would expect a faster reaction from PUT, but we need to take into consideration that a higher concentration of CAD was used, so a faster reaction is justified. The SPE reaction is slower than CAD and PUT, due to its even lower—almost negligible—vapor pressure (almost 0.00 kPa at 25 °C). 

Although the different vapor pressures affect the speed of reaction between the amines tested with the PCLCU1, the color of the material changes effectively as, in all cases, their pKa (Table S1) is above 9 and therefore they are present in their protonated form in the presence of water molecules, initiating thus the interaction with curcumin and its effective color change. In fact, as already mentioned, the color change is attributed to the reaction between the amines and the water to form protonated amines and OH^−^. This results in the formation of an alkaline environment close to the surface of the film, with the phenolic hydroxyl group of the curcumin to easily form an acid–base reaction with the OH^−^, resulting in the formation of the phenolic oxygen anion in the curcumin structure, which optically results in the observed color change from yellow to brown/reddish [78]. 

In the case of HIS, the color change in the mat is the slowest one, while instabilities on the performance have been observed. In fact, HIS is an aromatic molecule, with an imidazole ring attached to its ethylamine chain (Figure 2c), something that makes it more stable, with vapor pressure almost negligible in environmental conditions, and with the highest boiling point and evaporation enthalpy among the amines tested (Table S1 of Supporting Information), leading to a much slower evaporation process in the current experimental conditions. Moreover, the fact that the pK_a_ of the imidazole group is 5.88 indicates that, in the presence of water molecules, only the side chain is protonated (pK_a_ = 9.68), and this makes HIS less prone to react with curcumin. 

**Figure 4:**
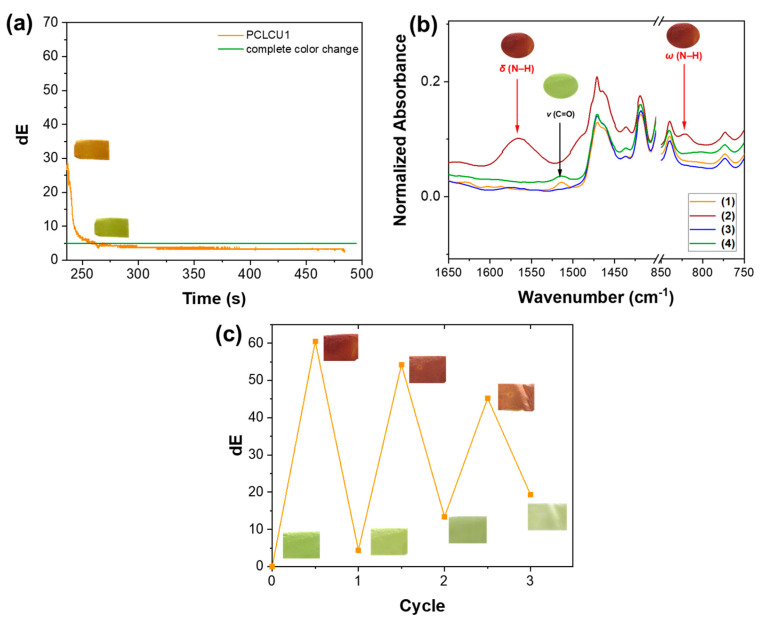
(**a**) The kinetics of the color recovery of the PCLCU1 after the exposure to TMA. the horizontal line indicates the dE = 5 (**b**) ATR-FTIR spectra of PCLCU1 (1) before exposure to CAD, (2) immediately after the exposure to 527.8 ppm of CAD, (3) 1 week after the CAD exposure (the sample was stored at ambient conditions in the dark), and (4) immediately after the CAD exposure and subsequent exposure to acid bath. In the insets, the color of the material with the presence of each peak. (**c**) The three cycles of CAD-HCl exposure of PCLCU1. In the insets, the color of the material after each exposure.

After the exposure of the mats to the amines, the cell was opened, in order to provoke the release of the vapors and the reorganization of the curcumin molecules resulting in the recovery of the sample’s color to the original yellow. Indeed, as shown in Figure 4a, after the interaction with the TMA, the material was able to spontaneously return to its initial yellow color in 259.0 ± 17.1 s after the cell opening, with the dE falling below 5 in 24.8 ± 10.5 s. This indicates that the TMA has a similar behavior with the DMA studied in our previous work [13] and thus the material is reusable with VAs.

However, the BAs-exposed PCLCU1 does not return to its initial yellow color upon exposure to the ambient atmosphere (Figures S5 and S11b of Supporting Information), indicating that in the case of BAs the color reversibility is not a spontaneous process. This can be attributed to the low volatility of the BAs with respect to the VAs and to the higher basicity of the molecules (Table S1 of Supporting Information) that makes their neutralization at ambient conditions more difficult. To demonstrate that the reaction of the BAs with the curcumin is not permanent and that the material can recover its initial properties (to return to the yellow color), and, thus, that it can be reused, the BAs-exposed samples were placed in HCl (1 M) liquid environment for 24 h. Using CAD as a representative BA, it can be seen in the Supporting Information Figure S11d, that the color of the PCLCU1 returns to yellow. 

ATR-FTIR spectra of the PCLCU1 mat after its exposure to CAD, but also after being left at ambient conditions in the dark for 24 h, showed that the typical peaks of the amine at 1564 cm^−1^ and 829 cm^−1^ attributed to the bending and wagging of N–H, respectively, are evident, confirming that the amine cannot be spontaneously desorbed from the sample under ambient conditions (Figure 4b(2)). However, the amine peaks disappear after the acid bath, proving that the assisted desorption of the amine is necessary (Figure 4b(4)). At the same time, the peak at 1511 cm^−1^, attributed to the stretching of C=O of the keto form of curcumin, disappears with the exposure of the material to CAD, during which the curcumin takes its enol form, and it appears again only after the acid bath. This implies that the keto form of curcumin is recovered. It should be mentioned that the loss of the peaks at 1564 cm^−1^ and 829 cm^−1^ is observed also one week after the exposure of the material to CAD, without the acid wash (Figure 4b(3)). However, in this case, the peak at 1511 cm^−1^ representative of the keto form, is not observed, indicating a possible modification of the curcumin, as also proved by the loss of its color (Figure S11c of Supporting Information). These results reveal a non-permanent physical interaction between the curcumin and the amines, which is reversible if the PCLCU1 is exposed to a more acidic environment, otherwise the curcumin’s properties are altered, probably because the enol form is less stable than the keto one, which has a more stable carbonyl group. Indeed, such phenomena of alkaline degradation of curcumin have been also observed in previous studies [24,79].

After the confirmation of the material’s ability to return to its initial yellow color with the assistance of HCl in liquid form, three *yellow-red-yellow* cycles were performed in the presence of CAD and, subsequently, of HCl vapors. From Figure 4c, it is obvious that the material significantly changes its color after 2 min of exposure to CAD vapors, reaching a dE_max_ of 60.5. The subsequent exposure of the sample to HCl vapors for ca. 2 h leads to the CAD desorption, and therefore to the color recovery of the mat, with a final dE_min_ of 4.4. As noticed from Figure 4c, the yellow-red change is always complete (dE > 5), but lower dE_max_ values appear in the second and third cycle (54.2 and 45.2, respectively). At the same time, regarding the red-yellow recovery, it is visible that the material turns back to a yellowish color, but not to the original one, before any exposure. The dE_min_ value is lower than 5 only after the first cycle, while for the second and third cycle dE_min_ is 13.4 and 19.3, respectively, with respect to the initial color of the non-exposed mat. Since the dE is a vector variable, the fact that it is higher than 5 during the return of the material to its yellow color does not mean that the material takes another color, but, as it can be optically confirmed from the insets of Figure 4c, the yellow hue is different from the original one (before any exposure). This, along with the lower dE_max_ values for cycles two and three, probably indicates that in each cycle, not all of the curcumin molecules return from the enol to their keto form, although the color change is always intense (Figure 4c). It is of note that curcumin tends to be less stable when in alkaline environments [80], so the prolonged stay of the curcumin in the enol form may have gradually led to the degradation of the material.

## 4. Conclusions

To sum up, in this work, the responsivity of porous PCL curcumin-loaded fibers to alkaline vapors generated by various amines was investigated. As demonstrated by CIELAB color space analysis, the color changes of the porous material upon exposure to the VAs and BAs can be visually perceived by both experienced and inexperienced users, even in the presence of low amine concentrations in the studied environment. Moreover, the mats were able to recover their initial yellow color after their exposure to the amines in an assisted way in the presence of acid vapors for three consecutive exposure cycles. Finally, yet importantly, the presence of curcumin provided the mats with antioxidant activity, enabling, upon physical contact, the amelioration of environments such as food packaging or wound bed by preserving the food or by reducing oxidative stress, respectively. All these results highlight the importance of such a multifunctional single composition system with high porosity for use either as an antioxidant system or as an indicator of amines, according to the needs of the user. Such materials could cover applications such as intelligent food packaging, biomedicine, and environmental protection where the real-time visual inspection of the potentially alkaline environment and/or antioxidant activity are necessary. For example, with future studies on the correlation of the color change to the presence of different amines in the same medium, an explicative color gradient around the indicator can be developed to relate the mats color change to the state of the selected environment.

## Data Availability

The data presented in this study are available on request from the corresponding author.

## Supplementary Materials

The following supporting information can be downloaded at: https://www.mdpi.com/article/10.3390/s23229288/s1, Figure S1: Chemical structures of curcumin; Figure S2: Vertical electrospinning set-up and the non-solvent induced phase separation method; Figure S3: SEM images of the PCLCU05 and PCLCU1 porous fibers; Figure S4: Experimental setup; Figure S5: PCLCU1 after its exposure to CAD; Figure S6: CIELAB kinetics curves and fitting for dE of PCLCU1 during TMA exposure and the fitting parameters; Figure S7: The types of sigmoid Boltzmann response-time curves; Figure S8: CIELAB kinetics curves and fittings for dE of PCLCU1 during exposure to BAs; Note S1: The PCLCU1 fibers’ detection limit of TMA, includes Figure S9: linear fitting dependence of dE_max_ to TMA vapors of different concentrations for PCLCU1 mats; Figure S10: CIELAB kinetics curves and fittings for dE of PCLCU1 during exposure to different concentrations of BAs; Figure S11: PCLCU1 before, during and after exposure to CAD without and with acid bath; Table S1: The boiling point, vaporization enthalpy, vapor pressure and pKa of the amines; Table S2: Amine concentrations used for exposure of PCLCU1, dE_max_, time for dE_max_, time for dE ≥ 5. References [23,81,82] are cited in the supplementary materials.
