# Supplementary material for: Functional Polymeric Membranes with Antioxidant Properties for the Colorimetric Detection of Amines
Source: Sensors (Basel). 2023 Nov 20;23(22):9288. doi: 10.3390/s23229288 (PMC10675507; doi:10.3390/s23229288)
Supplement: Supplementary file 1 [file sensors-23-09288-s001.zip › sensors-2663653.pdf]

Supplementary Materials

Functional Polymeric Membranes with Antioxidant Properties for the Colorimetric Detection of Amines

Despoina Kossyvaki [1,2], Matteo Bustreo [3], Marco Contardi [1], Athanassia Athanassiou [1] and Despina Fragouli [1,*]

[1] Smart Materials, Istituto Italiano di Tecnologia, Via Morego 30, 16163 Genova, Italy; despoina.kossyvaki@iit.it (D.K.); marco.contardi@iit.it (M.C.); athanassia.athanassiou@iit.it (A.A.)

[2] Dipartimento di Informatica Bioingegneria, Robotica e Ingegneria dei Sistemi (DIBRIS), Università degli studi di Genova, Via Opera Pia 13, 16145 Genova, Italy

[3] Pattern Analysis and Computer Vision, Istituto Italiano di Tecnologia, Via Enrico Melen 83, 16152 Genova, Italy; matteo.bustreo@gmail.com

* Correspondence: despina.fragouli@iit.it

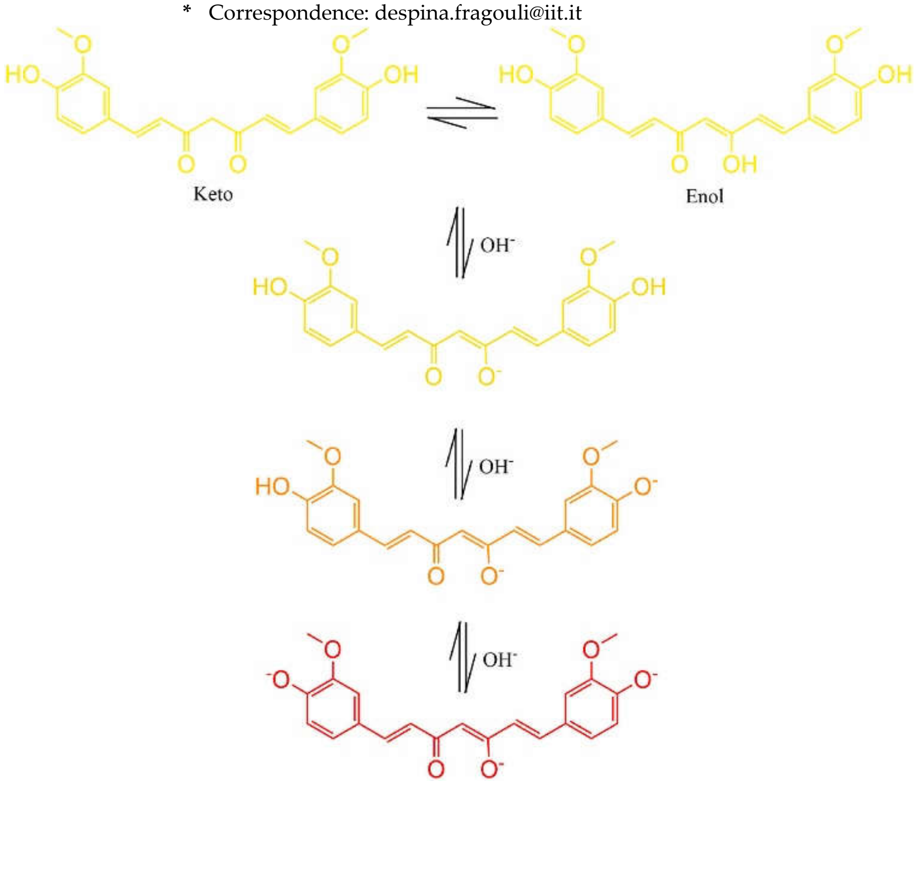

Figure S1. The keto and enol structures of curcumin. Reproduced with permission from [1].

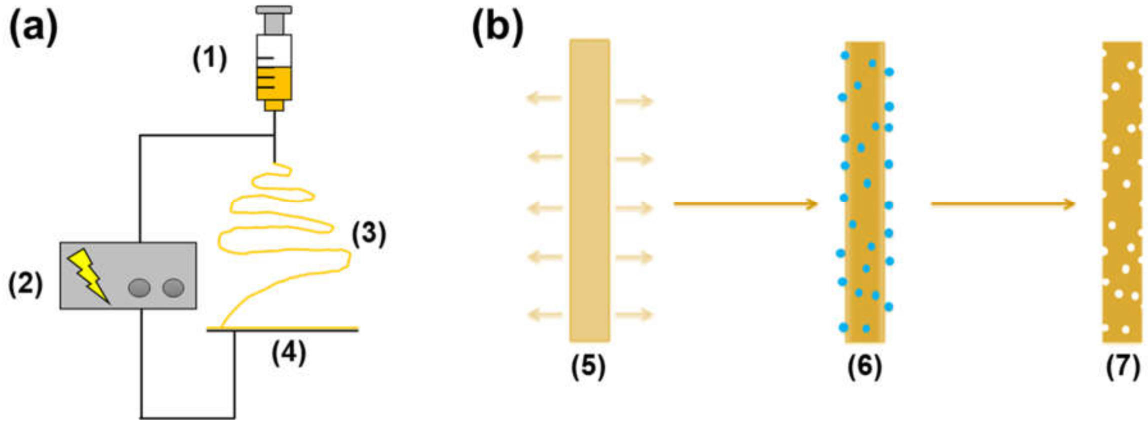

Figure S2. (**a**) Vertical electrospinning set-up with (**1**) the electrospinning solution, (**2**) the high voltage, (**3**) the fibers generation and (**4**) the collector. (**b**) The non-solvent induced phase separation method: the polymer solvent(s) with low boiling points evaporate immediately (**5**), leaving a polymer fiber with the non-solvent(s) with high boiling point (**6**), which subsequently evaporate, giving the fiber its final porous morphology (**7**).

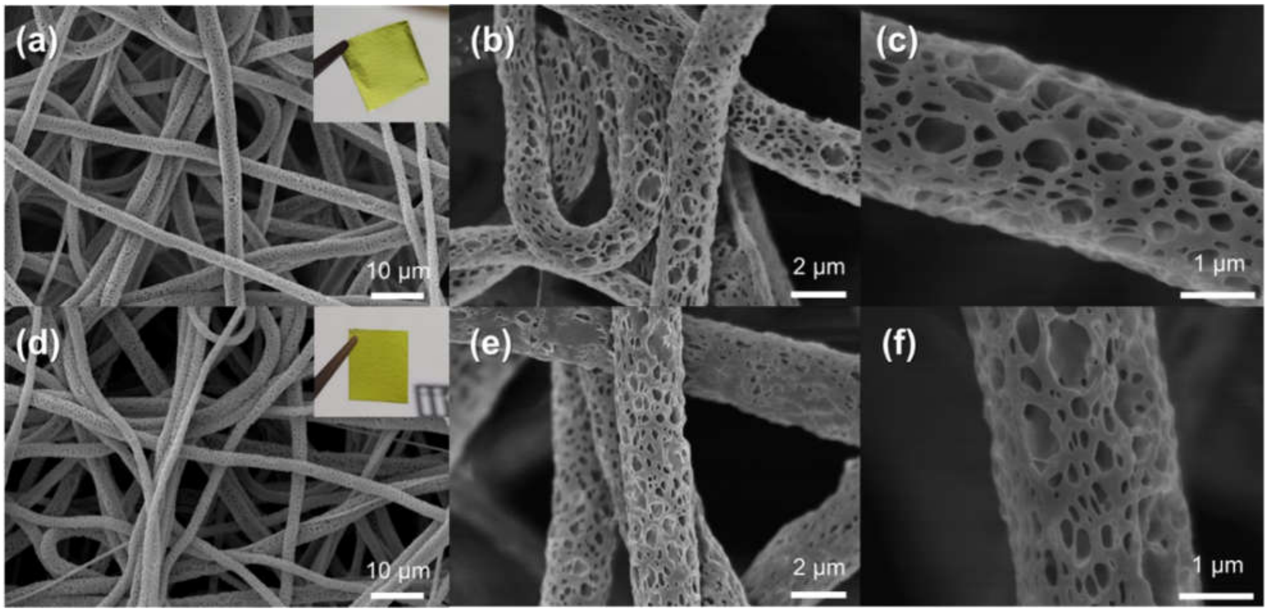

Figure S3. The curcumin-loaded (**a**–**c**) PCLCU05 and (**d**–**f**) PCLCU1 porous fibers in different magnifications. In the insets of (**a**,**d**) the images of the PCLCU05 and PCLCU1 fiber mats, respectively.

Table S1. The boiling point, vaporization enthalpy, vapor pressure, pK_{a1} and pK_{a2} of the studied amines, retrieved from: National Center for Biotechnology Information (2023), https://pubchem.ncbi.nlm.nih.gov/, and from [2,3]

Amine	Boiling point (ºC)	Vaporization Enthalpy (kJ/mol)	Vapor pressure (kPa) @ 25ºC	pK_{a1}	pK_{a2}	pK_{a3}
TMA	2.8±3.0	22.9±0.0	228.85±0.00	9.80	-	-
CAD	179.0±8.0	41.5±3.0	0.13±0.04	10.25	9.13	-
PUT	159.0±8.0	39.6±3.0	0.35±0.04	10.80	9.60	-
SPE	246.6±8.0	48.4±3.0	0.00±0.07	10.81	9.94	8.40
HIS	380.3±0.0	62.8±3.0	0.00±0.10	9.68	5.88	-

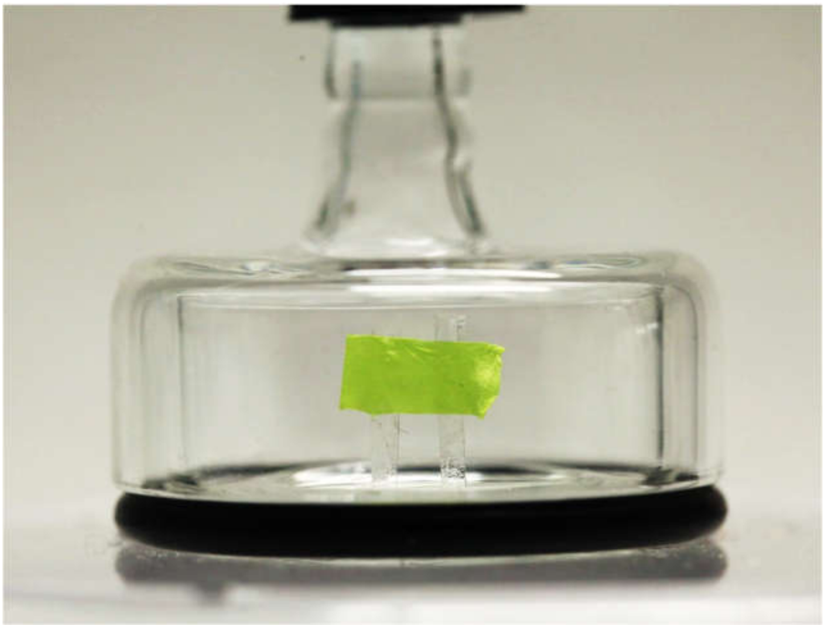

Figure S4. The homemade cell containing the PCLCU1 fibrous mat.

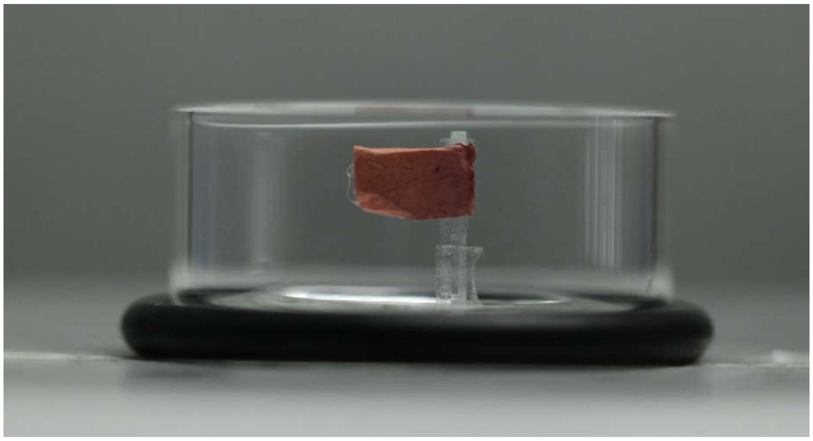

Figure S5. PCLCU1 after its exposure to CAD.

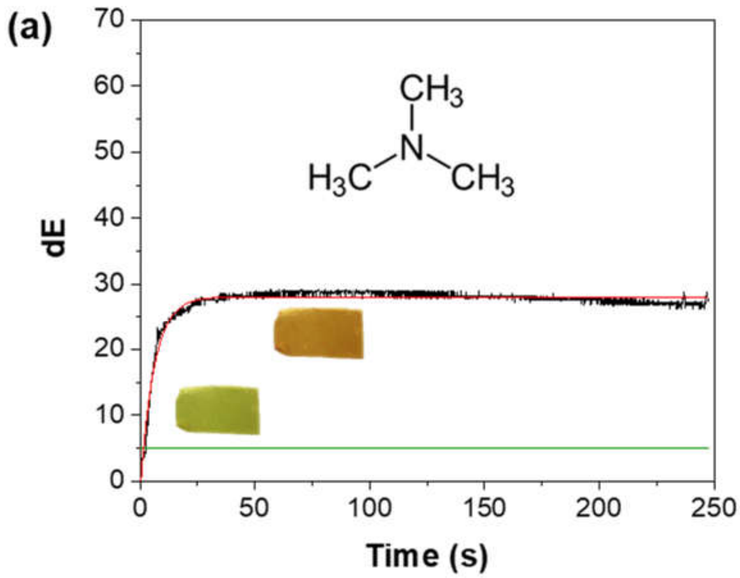

(b)

dE_{max}	t_0 (s)	dt (s)	R^2
27.6 ± 0.5	13.4± 11.6	0.1 ± 0.0	0.97± 0.01

Figure S6. (**a**) The dE evolution of the PCLCU1 mat over time, and its fitting using the pseudo-first order kinetics equation, during the exposure to TMA. Inset: the color of the mats before and after exposure, and the TMA molecular structure. The red line represents the fitting and the black one the original data. The horizontal line indicated the dE=5. (**b**) The fitting parameters of the pseudo-first order kinetics.

Table S2. The concentrations of the amines used for the exposure of PCLCU1, the dE_{max} values, the time needed for the mats to reach dE_{max}, the time needed for the mats to reach $dE \geq 5$, and the fitting parameters of the sigmoid curves (t_0, dt and R^2).

Amine	Concentration (ppm)	dE_0	dE_{max}	t_{dEmax} (s)	$t_{dE=5}$ (s)	t_0 (s)	dt (s)	R^2
**TMA**	244.8	0.0 ± 0.0	27.8 ± 0.5	59.5 ± 8.8	2.8 ± 0.5	13.2 ± 5.7	3.2 ± 5.7	0.95 ± 0.01
**CAD**	527.8	0.0 ± 0.0	66.1 ± 1.2	205.2 ± 46.6	2.5 ± 2.0	44.1 ± 9.2	9.5 ± 2.3	0.99 ± 0.01
	100.4	0.0 ± 0.0	49.3 ± 3.0	945.5 ± 203.8	155.0 ± 75.0	344.8 ± 21.8	37.2 ± 15.3	0.98 ± 0.03
	11.9	0.0 ± 0.0	43.0 ± 0.1	11689.2 ± 9490.8	1245.0 ± 105.0	1639.1 ± 510.5	631.8 ± 566.7	0.96 ± 0.03
**PUT**	504.0	0.0 ± 0.0	60.8 ± 0.4	458.5 ± 51.7	22.2 ± 1.7	86.0 ± 8.0	22.2 ± 1.7	0.99 ± 0.01
	100.8	0.0 ± 0.0	41.4 ± 1.8	1134.4 ± 635.3	195.0 ± 55.0	319.7 ± 117.0	62.7 ± 30.5	0.98 ± 0.01
	11.8	0.0 ± 0.0	45.2 ± 1.5	8468.0 ± 6975.3	2265.0 ± 255.0	2183.6 ± 2004.5	404.7 ± 321.1	0.96 ± 0.03
**SPE**	588.6	0.0 ± 0.0	53.1 ± 5.8	494.6 ± 6.8	24.9 ± 6.8	117.0 ± 26.0	31.6 ± 2.3	0.99 ± 0.03
	100.8	0.0 ± 0.0	46.3 ± 3.9	6330.0 ± 150.0	660.0 ± 480.0	2341.2 ± 540.1	553.9 ± 159.8	0.99 ± 0.03
	11.5	0.0 ± 0.0	43.8 ± 3.0	33630.0 ± 3150.0	240.0 ± 60.0	1279.9 ± 773.9	2564.4 ± 773.9	0.86 ± 0.08
**HIS**	689.2	0.0 ± 0.0	11.1 ± 3.8	21891.9 ± 13748.1	1080.0 ± 360.0	8307.6 ± 6541.4	4021.0 ± 3585.0	0.92 ± 0.07
	100.1	0.0 ± 0.0	10.2 ± 3.3	48270.0 ± 6930.0	48210.0 ± 6990.0	38502.96 ± 22324.9	16639.7 ± 1624.8	0.91 ± 0.05
	11.5	0.0 ± 0.0	17.4 ± 1.8	35880.0 ± 0.0	2880.0 ± 0.0	19792.1 ± 15570.6	6936.2 ± 4638.0	0.96 ± 0.01

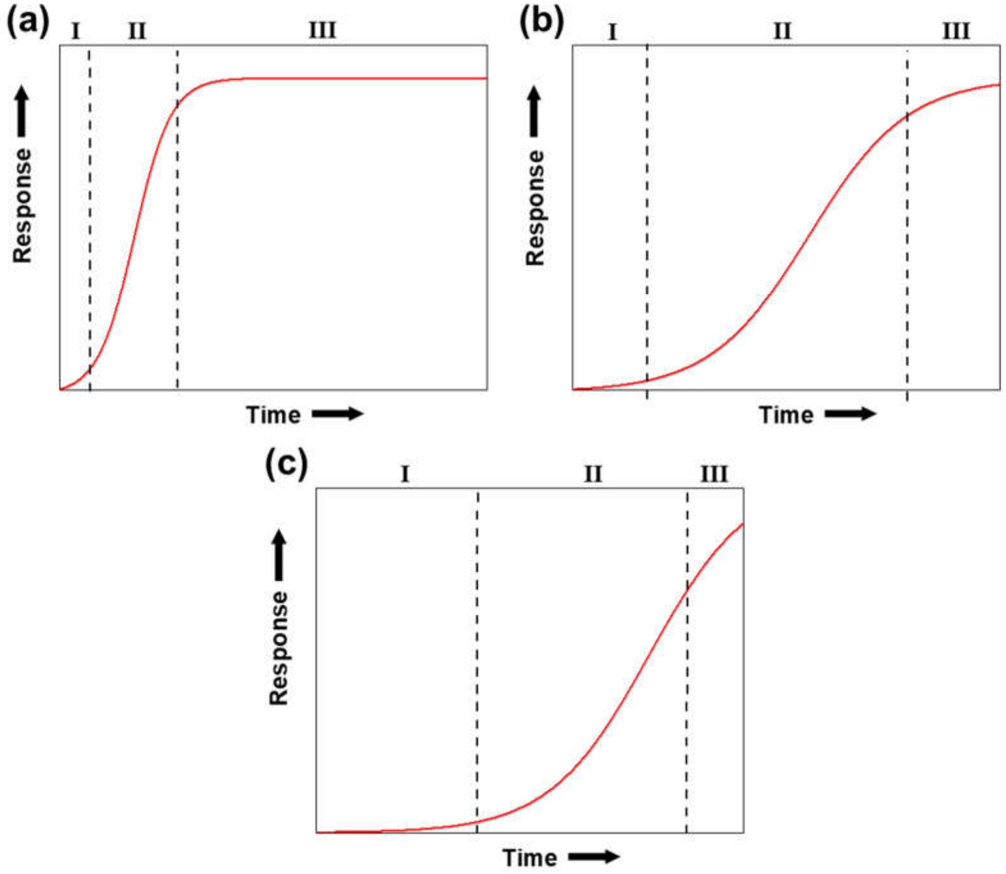

Figure S7. The (a) right-skewed, (b) symmetric, and (c) left-skewed sigmoid Boltzmann response-time curves.

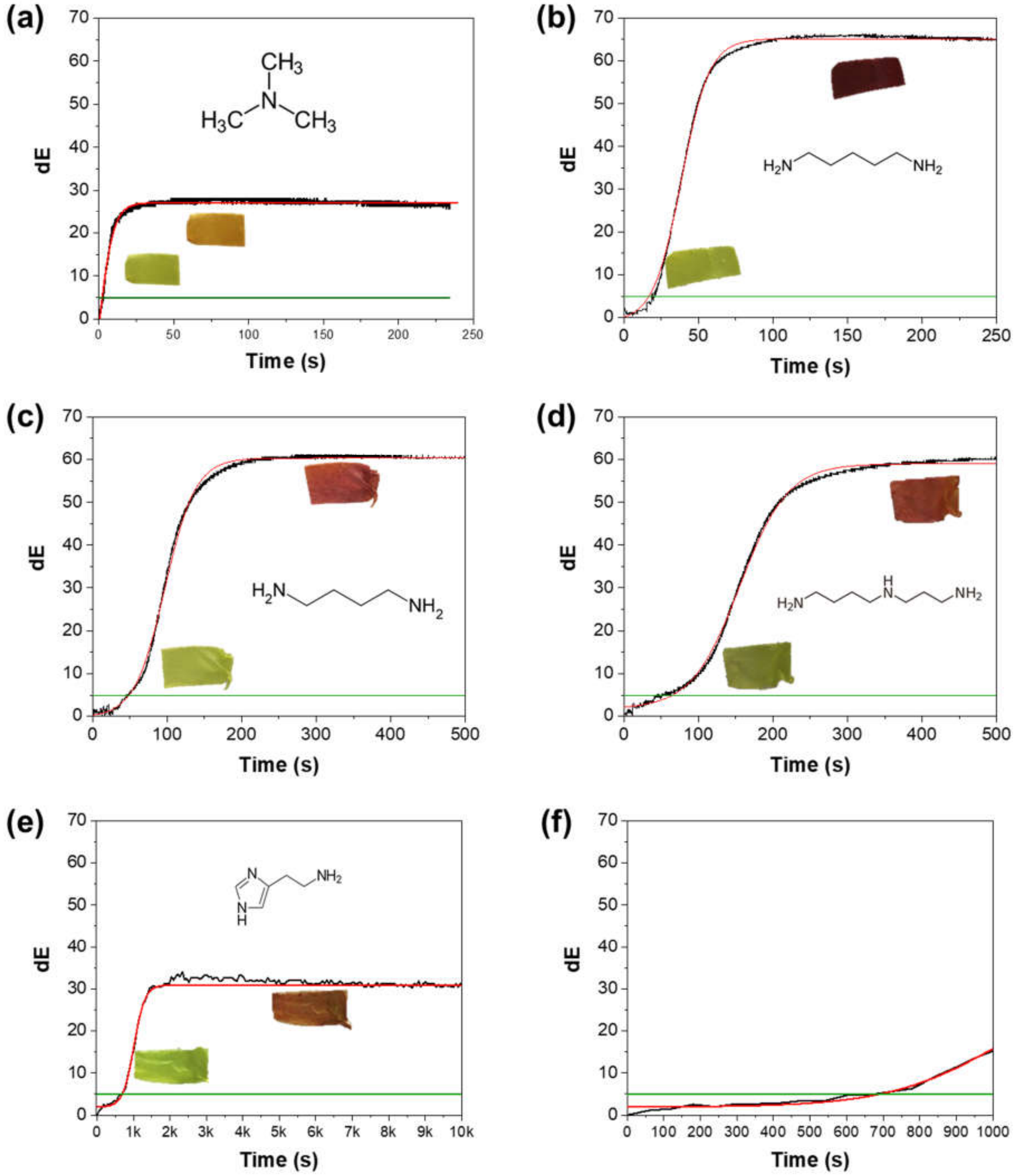

Figure S8. The dE evolution of the PCLCU1 mat over time, and its fitting using the Boltzman equation, during the exposure to (a) TMA, (b) CAD, (c) PUT, (d) SPE, and (e) HIS vapors. (f) The dE during the first 1000 s of exposure to HIS. In the insets, the

molecular structures, and the samples before and during the exposure to the amine vapors. The red line represents the Boltzmann fitting and the black one the original data. The green line indicates the dE value for the complete color change of the sample (dE=5).

Note S1.

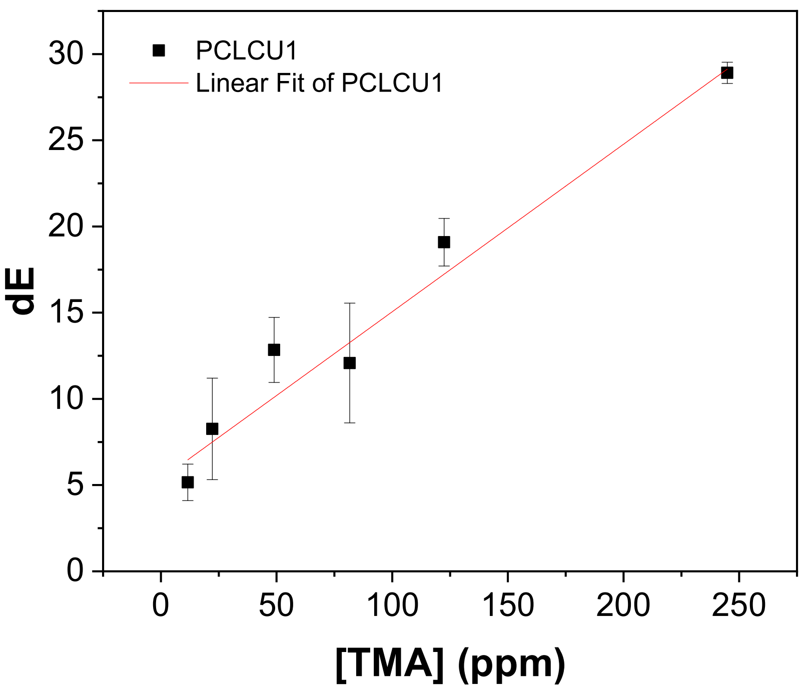

Figure S9. The linear fitting of the dependence of the dE_{max} to TMA vapors of different concentrations for the PCLCU1 mats ($R^2 \geq 0.98$).

The linear fitting of the data points was performed using the equation (S1):

$$dE = b \times [TMA] \tag{S1}$$

The fitting equation for the PCLCU1 is:

$$dE = 5.77 \times [\mathrm{T}MA]$$

Using this equation, the minimum concentration of the TMA in order to obtain dE=5 is 0.9 ppm.

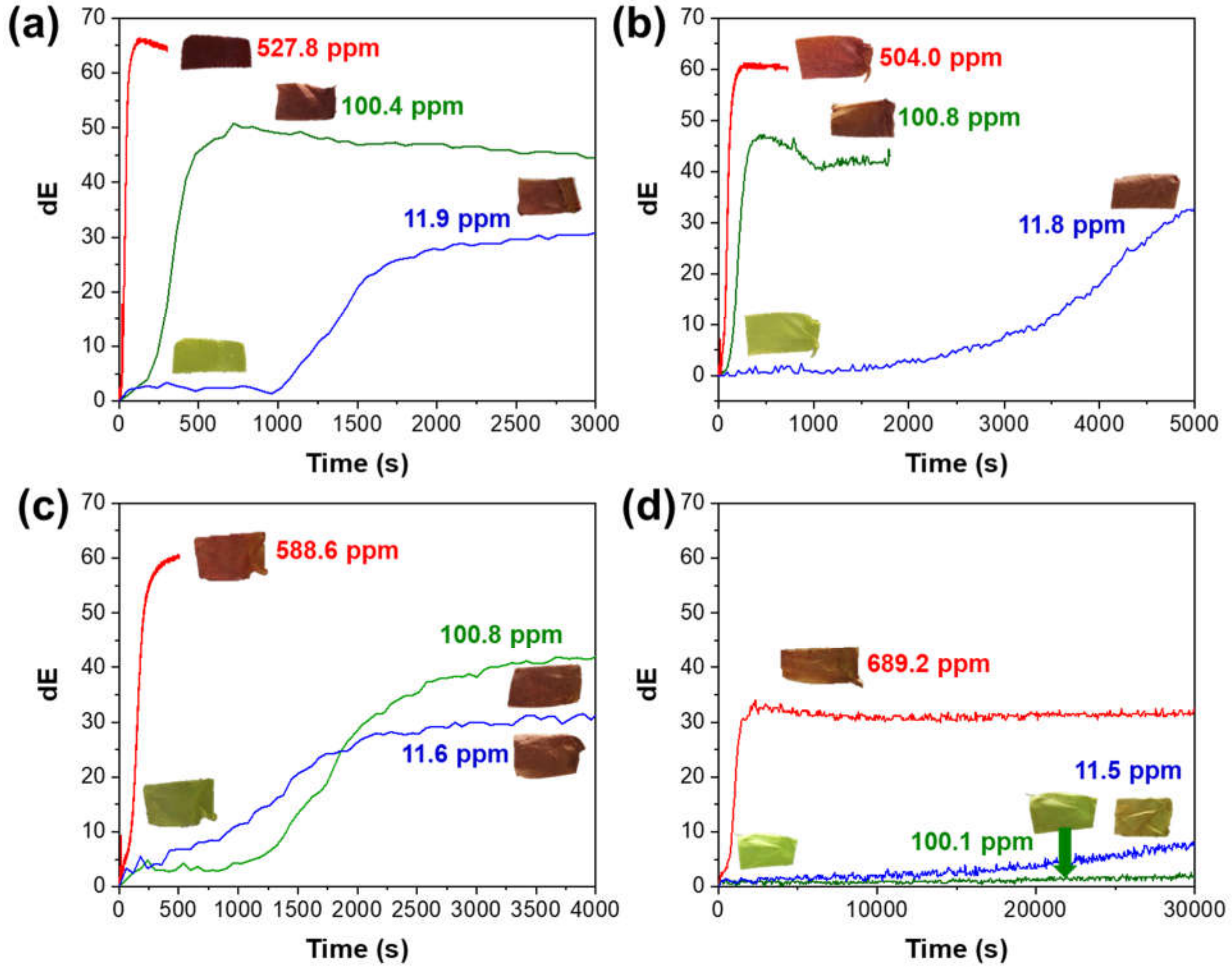

Figure S10. The dE evolution of the PCLCU1 mat over time during the exposure to different concentrations of BAs: (a) CAD, (b) PUT, (c) SPE, (d) HIS. On the insets, the images of the mats before and during the dE_{max} each exposure.

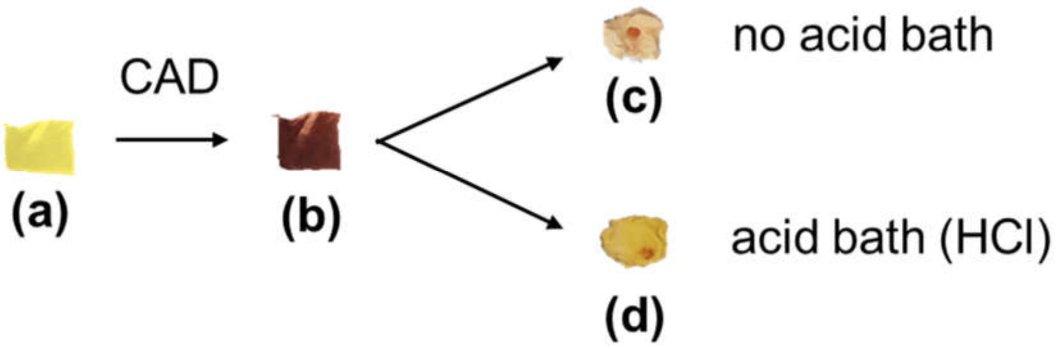

Figure S11. PCLCU1 (a) before exposure to CAD, (b) immediately after exposure to 527.8 ppm of CAD, and (c) one week after the CAD exposure (the sample was stored at ambient conditions and in the dark), and (d) immediately after the CAD exposure and subsequent exposure to acid bath.

References

1. Gonçalves, J.; Filho, D.O.; Romanelli, M.; Bertolo, V.; Alison, M.; Rodrigues, V.; Aparecida, C.; Silva, C.; Campos, F.; Odoni, A.; et al. Curcumin: A Multifunctional Molecule for the Development of Smart and Active Biodegradable Polymer-Based Films. *Trends Food Sci. Technol.* **2021**, *118*, 840–849, doi:10.1016/j.tifs.2021.11.005.

2. Nahi, O.; Kulak, A.N.; Zhang, S.; He, X.; Aslam, Z.; Ilett, M.A.; Ford, I.J.; Darkins, R.; Meldrum, F.C. Polyamines Promote Aragonite Nucleation and Generate Biomimetic Structures. *Adv. Sci.* **2023**, *10*, 1–12, doi:10.1002/advs.202203759.

3. Izquierdo, C.; Gómez-Tamayo, J.C.; Nebel, J.C.; Pardo, L.; Gonzalez, A. Identifying Human Diamine Sensors for Death Related Putrescine and Cadaverine Molecules. *PLoS Comput. Biol.* **2018**, *14*, 1–20, doi:10.1371/journal.pcbi.1005945.
